# Changes in monthly unemployment rates may predict changes in the number of psychiatric presentations to emergency services in South Australia

**DOI:** 10.1186/s12873-015-0042-5

**Published:** 2015-07-24

**Authors:** Niranjan Bidargaddi, Tarun Bastiampillai, Geoffrey Schrader, Robert Adams, Cynthia Piantadosi, Jörg Strobel, Graeme Tucker, Stephen Allison

**Affiliations:** Mental Health Informatics Unit, Country Health SA, Flinders University, School of Medicine, Lvl 1, 22 King William Street, Adelaide, SA 5000 Australia; Flinders University, School of Medicine–Discipline of Psychiatry, Adelaide, Australia; Mental Health Informatics Unit, Country Health SA, Adelaide, SA 5000 Australia; The Queen Elizabeth Hospital, University of Adelaide, School of Medicine–Discipline of Medicine, Adelaide, SA Australia; Mental Health Observatory Research Unit, Country Health SA, Adelaide, SA 5000 Australia; Epidemiology Branch, SA Department for Health and Ageing, School of Medicine–University of Adelaide, Adelaide, Australia

**Keywords:** Mental health, Times series modelling

## Abstract

**Background:**

To determine the extent to which variations in monthly Mental Health Emergency Department (MHED) presentations in South Australian Public Hospitals are associated with the Australian Bureau of Statistics (ABS) monthly unemployment rates.

**Methods:**

Times series modelling of relationships between monthly MHED presentations to South Australian Public Hospitals derived from the Integrated South Australian Activity Collection (ISAAC) data base and the ABS monthly unemployment rates in South Australia between January 2004–June 2011.

**Results:**

Time series modelling using monthly unemployment rates from ABS as a predictor variable explains 69 % of the variation in monthly MHED presentations across public hospitals in South Australia. Thirty-two percent of the variation in current month’s male MHED presentations can be predicted by using the 2 months’ prior male unemployment rate. Over 63 % of the variation in monthly female MHED presentations can be predicted by either male or female prior monthly unemployment rates.

**Conclusions:**

The findings of this study highlight that even with the relatively favourable economic conditions, small shifts in monthly unemployment rates can predict variations in monthly MHED presentations, particularly for women. Monthly ABS unemployment rates may be a useful metric for predicting demand for emergency mental health services.

## Background

With Europe experiencing a major economic downturn accompanied by marked increases in unemployment, there have been concerns raised about the impact on population mental health and suicide rates [1–6].

Families and communities experience poorer mental health from higher rates of unemployment [7, 8]. Generally, the negative impact of unemployment on mental health is more evident among men than women [9]. At a sociological level an increase in unemployment rate is a marker of social fragmentation and thereby contributes to mental health issues [10]. This association has been long established, and in 1897 Durkheim [11] argued that employment protected an individual against suicide by better integrating the individual with their society. Australia, unlike most Western nations, did not go into recession following the Global Financial crisis in 2007. However, there was an economic slow-down with an accompanying rise in unemployment from a low of 4.0 % in early 2008 to a peak of 5.8 % in late 2009 before declining to 5.0 % in April 2012 [12]. Emergency Departments (ED) in Australia have significant numbers of patients presenting with mental health problems as their primary problem, with this group accounting for between 4.5 and 5.6 % of all ED presentations [13]. There is evidence most people who receive Emergency Department Mental Health (MHED) care are unemployed [13] and ED might be their preferred pathway to crisis care during economic slowdowns.

While time-trend analysis has been used to analyse associations between unemployment and suicide [1], no study has used this method to analyse possible links between unemployment and MHED presentations. We were interested to determine whether in a country with relatively low levels of unemployment, the number of MHED presentations was associated with unemployment rates and whether this varied by gender. In this study, we analysed the effects of changes in rates of unemployment in South Australia on numbers of MHED presentations using a time-trend analysis between 2004 and 2011.

## Methods

### Data sets

Seasonally adjusted monthly unemployment rates for South Australia from January 2004 to December 2011 were sourced from ABS (Catalogue 6202.0–Labour Force, Australia, May 2012) [14]. The total number of people presenting to South Australian tertiary hospital EDs during the same period were sourced from the SA Health database, ISAAC, (Integrated South Australian Activity Collection). In South Australia, there are no separate psychiatric emergency departments and all mental health/psychiatric emergency presentations occur at general hospital emergency departments. All presentations with ICD codes in the range F00–F99 were categorized as psychiatric ED presentations. Gender was determined from demographic variables of each presentation. The rest were categorized as physical illness presentations.

The number of mental health presentations to ED was organized into 3 outcome series–all mental health presentations, female mental health presentations and male mental health presentations. Each of these time series was seasonally adjusted to remove seasonal variations of ED presentations using the ratio-to-moving-average Seasonal Decomposition method. All subsequent analysis was then performed on seasonally adjusted series.

### Ethics statement

As data was extracted from de identified administrative data sets, ethics approval was not sought.

### Analysis

The analysis involved a combination of cross correlation and ARIMA modelling. The first step of analysis involved a series of cross correlation computations between predictor (total unemployed, male unemployed and female unemployed) and outcome (MHED total presentations, male MHED presentations, female MHED presentations) time series variables using “arima” and “ccf” from the package “stats” in R 2.13 (http://www.r-project.org/). The residuals of both series were used as input for the cross-correlation analysis. Cross correlation coefficients between output series and input series with a lag k at time t were considered significant if the correlation coefficients were outside the standard error margin of 0.2.

We then used the ARIMA function in the SPSS (Chicago, IL, USA) Forecasting Module to derive a series of ARIMA models with unemployment as the predictor variable and mental health ED presentations as the outcome variables, by specifying auto regression (AR) with the lags that were shown to be significant in cross correlation analysis, with either undifferenced or first differenced Integration (I) components and Moving Average (MA) components of either [0] or [1,2]. We selected the best model of the above.

Automatic detection of outliers was made and the outliers were modelled accordingly, thus trimming was not performed. The stationary R-squared measurement determined was used as an indicator of goodness of fit for the models. The Ljung-Box Q provided the diagnostic statistics to check the presence of structure in the observed series that was not accounted for by the model. Significance values less than 0.01 were considered indicating the presence of structure in the observed series, which was not accounted for by the model; therefore, we ignored the model if it had a significant value less than 0.01. The Bayesian Information Criteria (BIC) is also reported as a comparative goodness of fit measure for each of the models derived. We also used SPSS version 19.0 Expert Modeler (Chicago, IL, USA) to automatically determine the best-fitting univariate ARIMA models for each of the outcomes time series variables that had shown significant cross correlation with predictor variables.

We compared the metrics of the models using unemployment as predictor variables with the best-fitting univariate ARIMA model derived by the Expert Modeller.

## Results

### Unemployment and physical illness presentations to ED

**Fig. 1:**
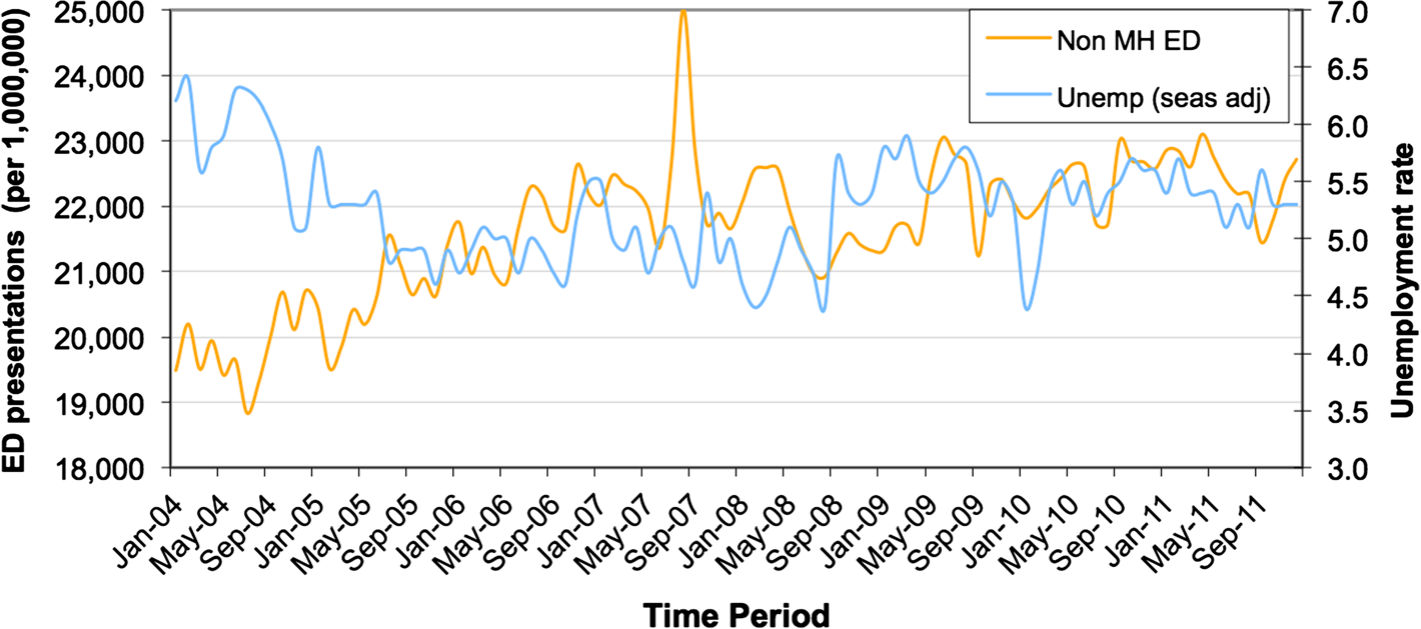
Unemployment rate (percentage) and physical illness presentations (per million) to ED per month for the duration of the study

We initially looked at associations between unemployment rate and physical illness presentations to ED (see Fig. 1). A cross correlation of prewhitened data showed no significant correlation between these two time series for varying lags of unemployment.

### Unemployment and mental health presentations to ED

Non-mental health ED presentations ranged from 22,450 to 33,697 per month (Mean 28,282.8; SE 234.40). MHED presentations ranged from 978 to 1421 per month (Mean 1226.5; SE 10.11). Table 1 shows the MHED diagnostic groups with affective disorders being the most frequent grouping.

**Table 1:** Mean MHED presentations per month for ICD-10F code diagnosis groups

ICD 10 diagnosis groups	Mean	SD
F00–F09: Organic including symptomatic, mental disorders	69.6	12.2
F10–F19: Mental & Behavioural disorders due to psychoactive substance use	296.2	63.0
F20–F29: Schizophrenia, schizotypal & delusional disorders	196.3	22.3
F30–F39: Mood (affective) disorders	156.6	35.2
F40–F48: Neurotic, stress related and somatoform disorders	402.1	38.5
F50–F59: Behavioural syndromes associated with physiological disturbances and physical factors	10.4	3.9
F60–F69: Disorder of adult personality and behaviour	42.2	11.5
F70–F79: Mental retardation	1.4	0.8
F80–F89: Disorders of psychological development	2.2	1.5
Unspecified mental disorder	51.0	13.8

**Fig. 2:**
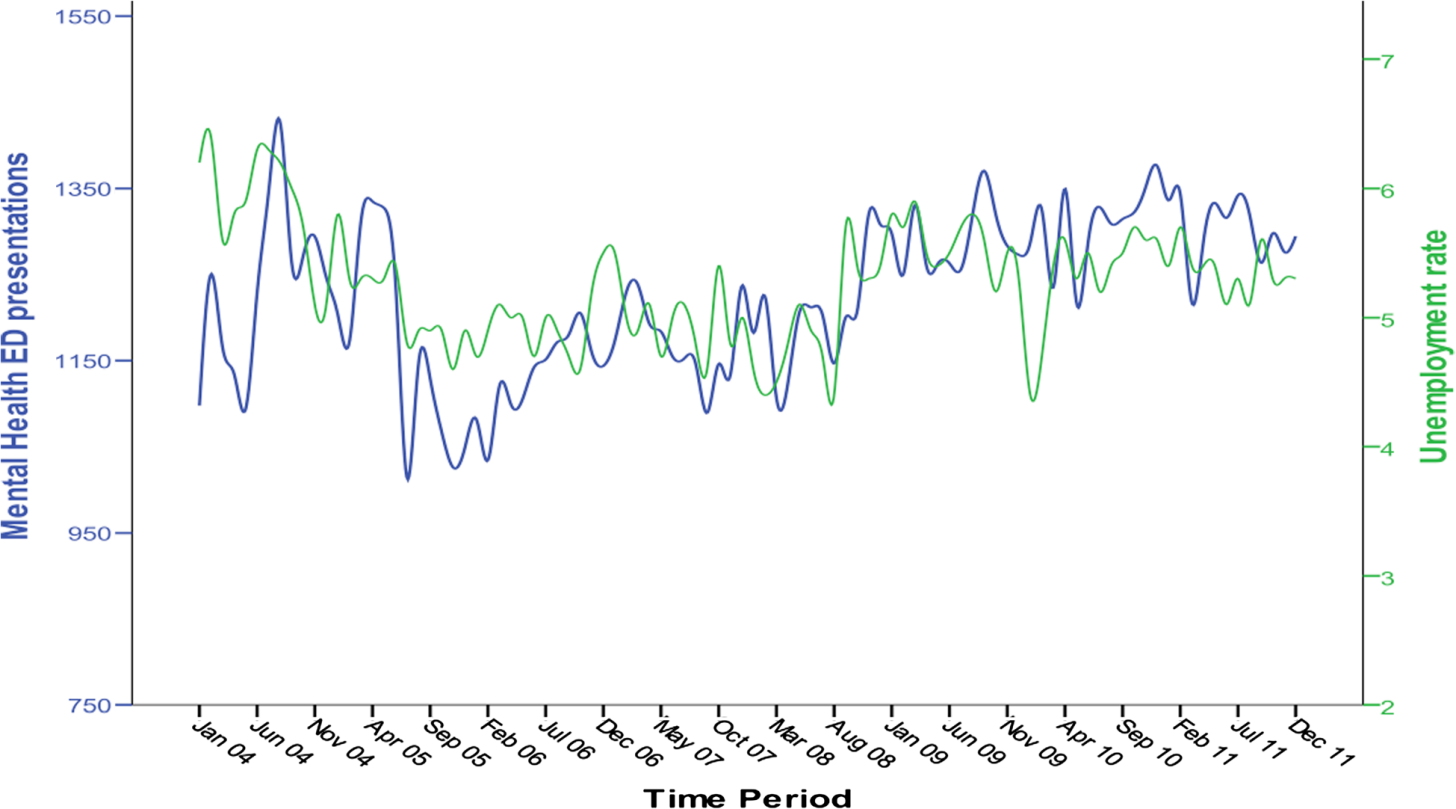
Number of Mental Health ED presentations (*blue line*) and unemployment rate (percentage) by month (*green line*) for the duration of the study

We initially used the SPSS Expert modeller to determine the best univariate ARIMA model for predicting Mental Health ED presentations. The best univariate model was ARIMA (0,1,3) with stationary-R-squared of 0.36 and Ljung-Box *p* = 0.70, and lowest BIC = 7.81. We then examined any association between total MHED presentations and unemployment rate (Fig. 2). A cross correlation of prewhitened series showed significant correlation between the unemployment and presentations, at current month (Correlation coeff = 0.22 (SE = 0.2)) and a lag of 2 months (Correlation coeff = 0.36 (SE = 0.2)).

We then derived transfer function ARIMA models with MHED presentations as the dependent variable and unemployment as a predictor variable. We tested several different models by specifying lags of 0 and 2 months as the AR, and varying the combinations of I = 0 or 1 and MA = 0 or [1,2]. The best model was ARIMA (AR = 0, I = 0, MA = [1,2]) that reached the highest stationary R-Squared of 0.69, Ljung-Box *p* = 0.19 and also the lowest BIC of 8.20. This analysis demonstrated a significant improvement in explaining variations of Mental Health ED presentations was achieved by introducing unemployment as a predictor variable, the stationary R-Squared improving from 0.36 to 0.69.

### Effect of gender

#### Male mental health presentations to ED

We then looked at the effect of gender. The best univariate model for predicting male MHED presentations was an ARIMA (0,1,1) that had stationary R-squared of 0.27 and Ljung-Box *p* = 0.16, BIC = 7.26.

We repeated cross-correlations analysis and observed significant associations between male unemployment rates and MHED presentations of males, most significantly with 2 months prior male unemployment rates (corr coeff = 0.36 (SE = 0.2)). The best ARIMA model using male unemployment rate with lag 2 as the predictor variable was an ARIMA (AR = 2, I = 1, MA = [1,2]) with stationary R-Squared of 0.32 and Ljung-Box *p* = 0.13, BIC = 7.42. This analysis shows that by introducing a male unemployment predictor variable with a lag of 2 months, for male MHED presentations, the stationary R-Squared was improved from 0.27 to 0.32.

A cross correlation analysis between female unemployment and male MHED presentations showed no significant association.

#### Female mental health presentations to ED

The best univariate model for predicting female MHED presentations was a simple univariate model that had stationary R-squared of 0.17 and Ljung-Box *p* = 0.05, BIC = 7.27. The cross-correlation analysis between female unemployment rates and female MHED presentations showed significant correlations with both current (Corr Coeff = 0.23; SE = 0.2) and previous months (Corr Coeff = 0.29, SE = 0.2) female unemployment rates. The best ARIMA model for predicting female MHED presentations using current month’s female unemployment rate as the independent predictor variable was (ARIMA (0,0,0)) with a stationary R-Squared of 0.63 and Ljung-Box *p* = 0.37, and lowest BIC = 7.00. By using the previous month’s female unemployment rate as predictor variable the best model observed was ARIMA (1,1,0) with stationary R-Squared of 0.50 and Ljung-Box *p* = 0.48, BIC = 7.20.

**Fig. 3:**
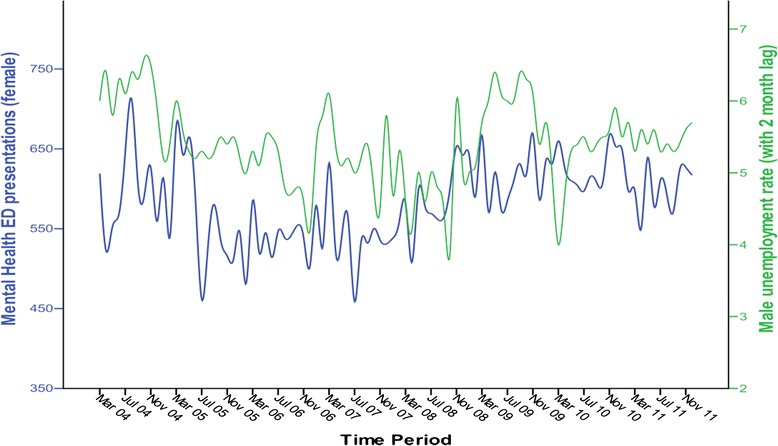
Number of female Mental Health ED presentations (*blue line*) and male unemployment rate (percentage) with 2 month lag, per month (*green line*) for the duration of the study

We also examined whether there was an impact of male unemployment on female MHED presentations. A cross correlation showed significant correlations between the female presentations and lag 2 of male unemployment (CorrCoeff = 0.24, SE = 0.2). The best ARIMA models for predicting female MHED presentations using 2 months’ prior male unemployment rate as the independent predictor variable was an ARIMA (2,0,0) model with a stationary R-Squared of 0.64 and Ljung-Box *p* = 0.95, BIC = 7.10 (Fig. 3).

This analysis demonstrated that by introducing either the current or previous month’s female unemployment rate or 2 months’ prior male unemployment rate as a predictor variable, a significant improvement was achieved in the ability to predict female MHED presentations.

## Discussion

Our findings suggest that unemployment might be an important social determinant increasing the number of visits to the medical emergency room for reasons of mental health. Unemployment rates were predictive of MHED presentations for both men and women in South Australia. Most of the association between unemployment rates and MHED presentations, however, was attributable to changes in ED use by women. While there is no clear evidence from life-events literature [15] that women are more sensitive than men to adverse life-events like unemployment, it is well established that women are more likely to seek help [16]; women have higher rates of depression [17]; and there is an over-representation of women with nonfatal suicidal behaviour presenting to EDs [18]. Women show more ability to empathize and this tendency might increase their liability for emotional symptoms in the presence of acute disappointments such as a family crisis arising from unemployment [19].

Before we address the possible policy implications of this study we need to consider several of its limitations. Firstly, correlation tests assess associations and do not establish causality. Thus, the association between unemployment and ED mental health presentations might be an indirect one, caused by other unidentified factors. However the study was carried out in an economy with close to full employment, so other factors related to economies in recession would not have confounded the associations found. Furthermore, while statistically significant, whether or not the effect of the association is clinically significant is not clear from our study. A further limitation relates to the fact that not all diagnoses used in the analysis were made by psychiatrists, some were made by ED physicians. Finally, the study was not designed to explore how presentation of different psychiatric diagnostic categories varied over time.

With an economy not in recession, these findings indicate that relatively small increases in unemployment rates might predict observed increases in emergency mental health demand. Unemployment data might therefore be useful for predicting the requirements for MHED service provision. Emergency responses may require more flexible health care and social service responses and funding arrangements. If further research confirms our finding then an argument for hospitals to plan ahead for increasing demand for MHED services when unemployment rates rise, akin to hospitals planning for increased admissions during wintertime could be developed [20].

There seems to be a critical time period of 1 to 2 months to plan ahead for changing demand. This short lag period suggest that effects of unemployment can be quite contemporaneous. However it is also likely that there would have been an anticipatory component preceding formal job loss. In terms of use of mental health services, a study in Illinois [21] conducted between 1970 and 1985 found a lagged effect of decreased labour force participation with increased psychiatric hospital admissions within 1 month. This parallels the lagged effect of 1–2 months noticed within South Australian EDs in our study. There is also evidence that unemployment leads to increased presentation to general practitioners [22].

## Conclusions

The findings from this study highlight that even with the relatively favourable economic conditions in Australia, small shifts in unemployment are associated with MHED presentations, particularly for women. Further studies should examine the effect of unemployment for spouses and on family systems since our study suggests that fluctuations in male unemployment can impact on MHED presentations for women.
